# Towards decolonising research methods training: the development of a locally responsive online learning course on research methods for mental health in war and conflict for researchers and practitioners in the Gaza Strip

**DOI:** 10.1017/gmh.2021.40

**Published:** 2021-11-16

**Authors:** Tamimi Nancy, Kienzler Hanna, Hammoudeh Weeam, Khalawi Hala, Regent Mathias, Giacaman Rita

**Affiliations:** 1 King's College London Ringgold Standard Institution – Global Health & Social Medicine, 40 Aldwych, London WC2R 2LS, United Kingdom of Great Britain and Northern Ireland; 2 Birzeit University Institute of Community and Public Health Ringgold Standard Institution, Ramallah, State of Palestine; 3 King's College London School of Social Science and Public Policy Ringgold Standard Institution – Global Health and Social Medicine London, London, United Kingdom of Great Britain and Northern Ireland

**Keywords:** Capacity building, decolonisation, Gaza Strip, mental health, online learning

## Abstract

**Background:**

Concerns exist that online learning directed at non-Western settings to strengthen research capacity imposes Western-centric epistemology, provides unidirectional transfer of knowledge, and neglects local paradigms and expertise. We argue that a plurality of voices, histories and epistemologies are essential to strengthen research capacity. We share our experience developing and teaching an online course for mental health professionals and researchers in the Gaza Strip.

**Methods:**

Birzeit University and King's College London developed and delivered the course equally, focusing on the intersection between qualitative research methods, mental health and conflict, and addressing local research needs. We incorporated local case studies and expertise, encouraged interaction in English and Arabic, and stimulated critique of Western theories. Seventeen participated, 12 completed the pre-course questionnaire, 15 completed the post-course questionnaire and four undertook semi-structured interviews.

**Results:**

Our pre-course survey showed participants most needed coding and qualitative data analysis skills. Post-course findings showed improved qualitative research skills. Most agreed the course was comprehensive and well delivered, with relevant case studies. Three themes were identified: (1) the course was locally contextualised and met students' needs; (2) the course fostered dialogic and multi-directional learning and (3) suggestions for improvements. Several participants wanted some topics in greater depth and further specialised training. A few suggested the course be in Arabic.

**Conclusion:**

Fostering multi-directional learning is key for non-Western knowledge, epistemologies, and languages to gain prominence in Western academia. A social transformation would see local researchers and educators engage with and use local methods and paradigms in mental health in war and conflict.

## Introduction

There are concerns around online learning initiatives directed at non-Western settings to strengthen research capacity. It is argued they impose Western-centric epistemology, provide unidirectional transfer of standardised knowledge, and neglect local paradigms, culture and expertise (de Sousa Santos, 2015; Adam, 2019; Gallagher and Knox, 2019; King *et al*., 2019). These approaches are perceived to reinforce colonial legacies of knowledge production, widen social injustices and overlook the value of different histories and epistemologies in forming a diverse global knowledge (Binka, 2005; de Sousa Santos, 2015; Adam, 2019; Gallagher and Knox, 2019; King *et al*., 2019). In contrast, scholars call for the decolonisation of knowledge as they question the assumption that Western European modes of thinking are universal and superior (Quijano, 2000). Instead, it is argued that decolonising knowledge has the potential to reach ‘social liberation from all power organized as inequality, discrimination, exploitation, and domination’ (Quijano, 2007, p. 178). According to Tuhiwai Smith, decolonisation is concerned with having ‘a more critical understanding of the underlying assumptions, motivations and values that inform research practices’(Smith, 2012, p. 24). Hence, decolonisation is a long-term process that involves divesting colonial cultural, linguistic and psychological legacies and fostering local knowledge production and documentation of indigenous struggles.

In this article, we share our experience of offering a collaborative online course aimed at strengthening research capacity among mental health professionals and researchers (MHPR) in the Gaza Strip (GS) in the occupied Palestinian territory (oPt), and seeking to decolonise knowledge. Our course, ‘Research Methods for Mental Health in War and Conflict’, was developed and delivered by researchers from the Institute of Community and Public Health (ICPH) at Birzeit University (BZU) in the oPt and King's College London (KCL), as part of the project Research for Health in Conflict – Middle East and North Africa (R4HC-MENA). Here, we describe, evaluate and critique the methods we employed to integrate local knowledge and expertise to enable multi-directional learning, and present the participants' evaluation.

## Mental health in war and conflict: the case of the Gaza Strip

The excessive burden of mental illness among civilian populations in conflict settings (Steel *et al*., 2009; Luitel *et al*., 2013; Silove *et al*., 2014; Gammouh *et al*., 2015; Naja *et al*., 2016) urgently requires research to inform mental health policies (Charlson *et al*., 2016; Bosqui and Marshoud, 2018). In the oPt, it was found that the mental health of Palestinians is adversely affected by the violence and human rights abuse faced in the ongoing Palestinian-Israeli conflict since the West Bank (WB) and the GS fell under Israeli military rule in 1967 (Giacaman *et al*., 2009; Cordesman and Moravitz, 2005; Madianos *et al*., 2011; Dimitry, 2012; El Masri *et al*., 2013; WHO Assembly, 2019). Palestinians in the GS have been under siege since the early 1990s which has destroyed the Strip's economy (Smith, 2015). Gazans are denied free access to the WB and abroad, including for much-needed medical and other services. In addition to periodic attacks, Israel waged wars on the GS in 2009, 2012, 2014 and 2021, with thousands dead, injured, displaced and maimed (McCarthy, 2021).

As a consequence of the siege and ongoing attacks, mental health and psychosocial problems have been identified as significant public health challenges. WHO has reported that an estimated one in 10 people in the GS is affected by severe or moderate mental health disorders (WHO Assembly, 2019). A study of adolescents found 68.9% developed post-traumatic stress disorder (PTSD), 40% moderate to severe depression and 94.9% severe anxiety (Elbedour *et al*., 2007). Similarly, another study highlighted that 41% of children in the GS had PTSD. Of these, 20% suffered from acute levels of PTSD. The study estimated 305 195 children in the GS need urgent psychological, social and medical interventions (Altawil *et al*., 2008; El-Khodary *et al*., 2020).

While mental healthcare needs are high in the GS, the mental health system is weak. Only about 2% of an already underfunded health budget is earmarked for mental health by the Ministry of Health (MoH). The number of healthcare workers in mental health facilities and private practice is just 11.91 per 100 000 population, consisting of 0.25 psychiatrists, 1.6 other medical doctors, 4.8 nurses, 2.2 psychologists, 2.5 social workers, 0.5 occupational therapists and 36.4 other health or mental health workers (Saymah *et al*., 2015). Regarding staff training, it is estimated that 4% of the training for medical doctors was mental health related, in comparison to 7% for nurses, while non-doctor/non-nurse primary health care workers received no mental health training (Saymah *et al*., 2015).

As well as gaps in mental health training, there is a lack of systematic mental health research, and a need to strengthen the local mental health research capacity (Marie *et al*., 2016; AlKhaldi *et al*., 2018). This is partly because MHPR face systemic barriers to learning about, and training in, research methods, mainly because restrictions on civilian movement make it difficult for external educators to visit, and for Gazans to travel for training (Marie *et al*., 2016; Giacaman *et al*., 2018). This is also emblematic of other war-affected contexts where local researchers struggle to build research capacity due to insecurity, restricted and unsafe movement, language barriers, lack of finance and limited face-to-face training (Zaheer *et al*., 2015; Bosqui and Marshoud, 2018; Hameed *et al*., 2018). Consequently, there are few authors from low- and middle-income countries (LMICs) and war and conflict settings, writing about local mental health issues in international journals. Policy making is mainly based on evidence generated by high-income country (HIC) scholars and interventionists using Western research tools and interpretative frameworks (McKee *et al*., 2012; Bowsher *et al*., 2019; Sukarieh and Tannock, 2019).

## Online learning in contexts of war and conflict

To our knowledge, there is no specific mental health research training in GS as capacity building focuses mainly on mental health service delivery. Nevertheless, in the oPt, online learning forms an essential part of higher education. Palestinian universities developed joint online programmes with international co-operation, such as OpenMed and METHOD (Mikki and Jondi, 2010). However, online learning in the oPt faces a lack of the following: an online learning culture; infrastructure and availability of necessary technology in households due to high cost, and regular electricity cuts; needs assessment and evaluation surveys among potential learners; sustainable funding; proper accreditation and recognition; educators and the political support and interest in the pedagogic value of online learning and staff development (Mikki and Jondi, 2010; Khader and Abu-aisheh, 2012; Al-Sayyed and Abdalhaq, 2016).

Overall, evidence shows that online learning effectively builds learners' capacity, overcomes insufficient research capacity and reduces the brain drain (International Telecommunication Union, 2006; Zander *et al*., 2006; Dodani *et al*., 2012; Zaheer *et al*., 2015). Online learning initiatives provide active, flexible learning with an emphasis on applying knowledge and interaction by combining a mix of pedagogical components focusing on simulation, communication, leadership and mentoring (Mason, 2003; Aczel *et al*., 2008). Online learning courses have also been criticised for their Western-centric focus and unquestioned colonial legacy (Bockstael, 2017), and for their delivery via corporatised, Western, digital platforms which ‘embed coloniality through digital neo-colonialism’ (Adam, 2019, p. 366; Gallagher and Knox, 2019). This is further perpetuated by restricting learning opportunities to fee-paying learners; designing and delivering courses solely by Western-based educators and institutions; using unidirectional, standardised, Western educational approaches to diverse international learners; using only Western languages; and lacking content which reflects local needs and culture (Adam, 2019). Such approaches assume superiority of Western knowledge systems and overlook the value of incorporating local knowledge, epistemologies and experiences.

While designing and evaluating our online course, we were not only mindful of these challenges, but also aware we were not able to fully overcome them. This was despite the fact we aimed to promote a culture of critical thinking amongst participants, encouraged social interaction and dialogue, stimulated deeper awareness of the social, economic, cultural and political circumstances that shape people's lives and their role in creating social change. We further recognised the fundamental relationship between knowledge and power (Foucault, 2008) and the importance and impact of power imbalance in the educational process (Freire, 1984). While our aim was to advance a heterogeneous, global knowledge system reflecting local contexts (Bockstael, 2017; Vaditya, 2018; Adam, 2019; Gallagher and Knox, 2019), we acknowledge that we based our course content on Western epistemic knowledge systems, trying to render them culturally sensitive, while at the same time incorporating diverse paradigms and local knowledge in a reflective manner(de Sousa Santos, 2015; Adam, 2019; King *et al*., 2019). We are therefore cautious that simply adapting Western epistemologies to local realities might inadvertently contribute to perpetuating epistemic oppression whereby local knowledge is, once again, pushed to the margins rather than occupying a central place in knowledge generation and exchange.

## Methods

### Context

Our online course forms part of the R4HC-MENA project, an initiative funded over 4 years by UK Research and Innovation (UKRI), to strengthen research and policy capacity across four countries affected by conflict: oPt, Lebanon, Jordan and Turkey. As part of this project, our Mental Health and Conflict workstream aims to support interdisciplinary mental health research capacity, by providing training in mental health research methods and creating a sustainable community network for local researchers. Simultaneously, our aim is for UK researchers to gain an increased knowledge and understanding of the epistemology, methods and practices used by researchers in the Middle East and North Africa (MENA) region, integrating this into their worldview, future training and research.

Consistent with the capacity strengthening literature emphasising the importance of collaboration between academic institutions of HICs and LMICs (Curry *et al*., 2012; Thornicroft *et al*., 2012; Ng *et al*., 2016; Beran *et al*., 2017), our course was the result of a longstanding partnership between researchers from KCL and BZU. Course materials were developed collectively and equally by teams of academics, research assistants, students and online technicians from both institutions. Except the technicians, all contributors are authors of this article. WH, HH and RG are Palestinians living in the WB. NT is Palestinian British, HK is German, MR is Belgian, all reside in the UK. Connected through almost a decade of collegiality and friendship, this collaboration rests on trust, respect, two-way knowledge exchange and learning.

We set out to reflectively engage with pedagogical approaches, literatures and research, through which we shared an understanding that Western paradigms dominate and influence our work in differential ways. Confronting educational power is challenging, as the Western power-knowledge nexus (Foucault, 1977) is firmly seated in university settings internationally. We came to understand that international collaboration, no matter how equitable it intends to be, is not enough to decolonise pedagogies in ways envisioned by thinkers like Paolo Freire, who fought for a liberated education where those involved can influence political powers by developing their own perceptual and analytical tools (Freire, 2013). Nevertheless, we believe it is crucial to work towards a future which challenges current power dynamics by fostering locally-driven knowledge production and use. This could be achieved through longstanding international partnerships, based on equity and solidarity between academics with different experience and knowledge (Bleiklie and Powell, 2005). Our online course has been inspired by this vision.

### Development

The course itself took shape when our team from KCL and BZU designed and delivered a face-to-face course, ‘Research Methods for Mental Health in War and Conflict’, at BZU in 2018. Although the course was open to MHPR from WB and the GS, none of the Gazans accepted onto the course were granted permission by Israel to travel to the WB. To overcome this, and to meet the need for mental health research, we decided to deliver the course to Gazans online.

It took about a year to develop and deliver the online course. First, our team reviewed online capacity strengthening literature and publicly available syllabi, identifying a lack of online courses providing qualitative methods training for mental health in conflict settings. These reviews, in addition to the findings of our previously conducted Training Needs Assessment among local Palestinian researchers (Giacaman *et al*., 2018), and participants' feedback following the face-to-face course, provided a robust background to the online course design.

Next, we explored transforming our face-to-face course into an online course with King's Online Service. Although we opted to use the KCL service provider who has expertise and established service provision, our teams from both KCL and BZU collaborated at every development stage through in-person meetings, zoom meetings and online training. An instructional designer trained us in curriculum development, course architecture, good practice for online learning, plus technical and design process. Our priority was to ensure the highest quality, local contextualisation of content, and the relevance to potential participants, while taking into account logistical challenges like lack of electricity and internet broadband in GS. We would have employed a Palestinian online course developer, but, to our knowledge, the local expertise in technology-enhanced learning was limited in the early stages of our course development. Fortunately, such expertise has developed since COVID 19 (UNESCO, 2020).

A major consideration was maintaining equal partnership between KCL and BZU. To ensure KCL would not be the sole owner of the course, we opted against hosting it on the KCL internal KEATS platform, and partnered with the external, UK-based online platform, Future Learn (FL), instead. While FL has extensive experience in delivering online courses across the world, including the MENA region, we are reflexive of the problems inherent in using a corporatised, UK-based platform for further education. Firstly, FL is considered a global platform not only because it is accessible everywhere in the world (Future Learn, 2021a), but also because it is in English, which reinforces the notion that ‘to be global is to be Western’ (Adam, 2019, p. 366). Further, systemic inequalities are embedded in platforms such as FL, as they contribute to the privatisation of education and limit the production of courses to ‘top institutions’, which are mostly Western (Future Learn, 2021b). We are conscious that our choice of platform limits our agenda of decolonising education, rendering it ‘partial’ colonial (Bhabha, 1994; Adam, 2019). However, given the systemic barriers resulting from the ongoing military occupation of Palestine, including its tech and education sectors, locally developed online learning platforms are not readily available. Consequently, we resorted to FL, while maintaining close contact and discussions among the team.

### Recruitment and evaluation

For course promotion, we used BZU's strong institutional network in GS, advertised through local mental health and psychosocial support organisations, and posted on social media platforms. We received 50 applications from professionals and researchers with Diploma, Bachelor, Master's or Ph.D. degrees. The team created an application ranking sheet, using a scoring system evaluating the overall quality of each application, assessing if the applicant worked or studied in the mental health field, resided in Gaza, or could have accessed similar training through institutional affiliations. English competency was also assessed and justified as the course was delivered in English and it was important to communicate with English-speaking instructors. We are aware that many courses in the medical and health professions are taught in English in the GS, and that the majority of mental health professionals are competent in English. However, it disadvantaged potential candidates with less exposure to Western-style education. Thus, we are mindful that our approach reinforced unequal power dynamics whereby Western approaches to knowledge production and dissemination were privileged. Aware of this weakness, we are currently translating the entire course into Arabic.

The course was delivered in February 2020 with 17 participants (11 women, six men) (Table 1). Among them, 14 participants met the requirements to receive an achievement certificate. Twelve participants (eight men, four women) completed the pre-course questionnaire, and 15 (11 men, four women) completed the post-course questionnaire while four (two men, two women) participated in semi-structured interviews. The pre-course questionnaire asked participants to self-assess their qualitative research skills, state their training needs and highlight their expectations (see Appendix 1 for survey). The results showed participants wanted to learn and strengthen their qualitative analysis skills, specifying thematic and content analysis; ‘choose appropriate methods’, and achieve ‘minimal bias’; conduct research projects independently; and improve research skills, ‘especially during emergency and conflicts’.

**Table 1.** Demographics of course participants

Demographics	Group	Frequency (*n*=17)	Percentage (%)
Gender	Female	6	35.3%
	Male	11	64.7%
	25–29 years old	2	11.8%
	30–34 years old	5	29.4%
Age range	35–39 years old	1	5.9%
	40–44 years old	5	29.4%
	45–49 years old	3	17.6%
	50–54 years old	1	5.9%
	Ph.D.	3	17.6%
Educational level	Master's degree	11	64.7%
	Bachelor's degree	3	17.6%
	Managerial/administrative position^a^	5	29.4%
Profession/occupation	Managerial/administrative position^a^ and health professional^b^	5	29.4%
	Researcher and health professional^b^	2	11.8%
	Nurse	2	11.8%
	Assistant professor and health professional^b^	1	5.9%
	Lecturer	1	5.9%
	Non-governmental organisation	6	35.3%
	Governmental organisation	3	17.6%
Organisation	UNRWA	3	17.6%
	United Nations Agency	2	11.8%
	Higher education	2	11.8%
	Non-profit organisation	1	5.9%

a Managerial position includes General Directorate, Medical Officer, National Professional Officer, Head of Health Centre, General Manager, Director Assistant.

b Health professional includes Medical Doctor, Nurse, Registered Nurse, Psychologist, Social Worker.

At the end of the course, participants evaluated it by addressing the same topics in the baseline questionnaire for comparative purposes. Participants also rated their agreement/disagreement with statements that captured their course experience and wrote about what they liked or disliked about the course, the challenging components, topics that they would have liked to learn about but weren't taught, and suggestions for future improvements (see Appendix 2 for survey). Quantitative survey results were analysed using SurveyMonkey, an online application with inbuilt software that automatically analyses data.

The semi-structured interviews were conducted with course participants to further understand their perceptions on the pedagogical methods and materials, integrated local knowledge and experiences. Interviewees were also invited to suggest further online course improvements. The topic guide was created by team members from both universities. While a recruitment email was sent to all participants, only four agreed to participate. Unfortunately, the sample was small (four) and involved participants who were all highly satisfied with the course, which created a positive bias. We attempted to mitigate this bias by clearly identifying their reflections in the results section. The interviews were carried out by NT using English or Arabic language, whichever the interviewees preferred. Anonymity and confidentiality were discussed. Three interviews were conducted in Arabic and one in English. They were conducted via Zoom, audio-recorded, lasted approximately 30 min and were transcribed by a professional transcriber (see Appendix 3 for topic guide). The qualitative feedback and interview transcripts were coded inductively and mostly descriptively (Saldana, 2012), and analysed using thematic analysis as outlined by Braun and Clarke (2006). The analysis was performed by the first author in consultation with both teams at BZU and KCL.

Ethical approval for conducting the surveys and semi-structured interviews was first obtained from the ICPH Ethics Review Committee. The positive outcome was then officially accepted by the Research Ethics Board at KCL MRA-19/20-18045.

### Implementation

The course ran over 4 weeks. First, we introduced the course and educators, learning objectives, and certification. To progress from one week to the next, participants had to pass an end of week test. We offered flexible timing, so participants could start and progress at any time. To receive the certificate, they had to complete at least 90% of the course, and score 70% in the tests overall. Certificates were issued jointly by KCL and BZU.

The first week introduced concepts and theories of war, societal violence and health, social suffering, and the qualitative paradigm. The second week addressed qualitative research design. The third week focused on qualitative data collection. The fourth week addressed qualitative data analysis and presenting research findings. Sessions were co-designed and taught by academics from both universities. Delivery methods included articles, discussions, exercises, quizzes, videos and end of week tests. Participants received personalised feedback from the teaching team for most exercises (Table 2). We employed the following methods to integrate local expertise and knowledge:

- Case studies

**Table 2.** The delivery method for each week's topics

Type	Week 1	Week 2	Week 3	Week 4
^a^Articles	War, societal violence and health The effect of war and violence on mental health Social suffering – an anthropological approach Historical perspective on vulnerability What is a vulnerable population? Sources of vulnerability What is qualitative research? Use of qualitative research in mental health and conflict Research paradigms	Qualitative research design components – finding a research topic Conceptual context Developing a statement of purpose Formulation of the research questions From topic to question Research question checklist Qualitative sampling Sampling and recruiting vulnerable and hard-to-reach populations	Using observation in qualitative research Semi-structured interviews and constructing interview guide Conducting semi-structured interviews Methodological themes in research interview of individuals receiving mental health services Focus group discussions (FGD) Reflecting on the strengths and challenges of conducting a FGD	What is coding? Deductive *v*. inductive coding How to code data Common qualitative data analysis approaches Six phases of thematic analysis Theme *v*. code Reflexivity Communicating your research Poster presentation Oral conference presentation Infographics
Discussions	The strengths and weaknesses of trauma-focused and psychological approaches	Finding your research topic Explore the pros and cons of snowball sampling and using gatekeepers with regards to your vulnerable populations	The use of observation in mental health in war and conflict Methodological themes in research interview of individuals receiving mental health services	Reflexivity Overcoming public speaking anxiety Academic elevator pitch
Exercises	Exploring social suffering in the context of your work Are all war-affected populations vulnerable? Explore various sources of vulnerability	Developing your statement of purpose Sampling vulnerable populations in the context of war and conflict	Observing your surroundings – create your own interview guide Construct your own focus group guide	Let's code! Carrying on thematic analysis Identifying strengths and weaknesses of a poster Create your own infographic
Quizzes	Qualitative *v*. quantitative	Developing your research question	Reflecting on the strengths, weaknesses, challenges and techniques employed in the focus group discussion	
Videos			How to conduct an interview How not to conduct an interview How to conduct FGD	Elevator pitch
Tests	End of week test	End of week test	End of week test	End of week test

a Articles refer to text materials written by the educators of the online course.

Included were case studies reflecting the mental health situation in Palestine and that of other conflict settings such as Afghanistan, Turkey, Serbia and Bosnia.

- Video production

Teaching videos about conducting semi-structured interviews, focus group discussions and an academic elevator pitch were created with support from KCL and BZU online teams. Topics and conversations focused on themes relevant to the Palestinian context.

- Dialogic, open discussions

Open discussion threads were offered for topics, encouraging participants to share their professional and research experiences, with the option to communicate in Arabic and English. We monitored and engaged with discussions daily. Input by participants included providing feedback on the course content, sharing examples and experiences, asking questions and engaging in peer discussions. Here is one example where a learner reflects on ethical considerations in research:

Maintaining privacy and anonymity is quite important for victims of torture. Such [a] vulnerable group [is] sensitive and fragile, especially if the case is a woman or a child. As a researcher, I will ensure building a trust relationship with the case before requesting them to sign a consent form for observation purposes.

- Multi-directional learning

We regarded our learners as experts, and encouraged them to link their learning to their personal experience. Reflective exercises helped us learn from participants, e.g. participants discussed characteristics of a vulnerable population in oPt and obstacles of recruitment, and described and presented research topics linked to the local context, such as, ‘Psychological disorders during the war among displaced families living in shelters’, and ‘Mental health and coping mechanisms among the medical team of emergency departments in GS hospitals during 2014 war’.

- Critique of Western adopted theories

We encouraged participants to address the relevance and applicability of Western adopted theories to the local context, stimulating reflections and locally-derived concepts. The following exchange between a participant and educator is exemplary:

*Learner*: When we want to study suicide, we commonly use the interpersonal theory (…) The theory explains the suicidal behaviour based on three components: the perceived burdensome, the acquired capacity to suicide, and the thwarted belonging.

*Educator*: This is one of the most popular theories in suicidology and has been used widely in the literature. However, some scholars questioned if the theory explains all suicides everywhere; rejecting the idea of the ‘universal truths’ that can be applied to all suicides; and highlighting that suicide is a complex, multi-factorial phenomenon and a highly contextual phenomenon. I wonder if you think such a critique is warranted when researching suicide in Palestine?

*Learner*: I totally agree with you. There are several limitations to Joiner's theory that can be observed from the suicidal cases in Gaza. Here, the psychological component within the focus of Joiner's theory, is highly dependent on the economic and social factors, in addition to the impulsive behaviour.

## Results: course evaluation and feedback

### Pre-course survey compared to post-course evaluation

Our pre-course survey showed a gap between various research tasks participants considered important, and their self-assessed ability to perform these well. The biggest gap was coding and analysing qualitative data, followed by using data for planning projects, communicating research findings and accurate data record keeping (Table 3).

**Table 3.** Participants’ pre-course training needs identification

	Very important, important or moderately important Total = 10	Performed well or very well Total = 10	Training gap
1. Developing an appropriate research objective in the mental health field	9	8	1
2. Designing research projects as part of your work in mental health	9	8	1
3. Identifying ethical concerns around mental health research	9	10	1
4. Reviewing scientific/academic literature	9	9	0
5. Accurate and complete record keeping of data (e.g. organising data systematically, developing spread sheets)	10	6	4
6. Collecting and collating relevant research information	10	9	1
7. Collecting qualitative data (e.g. interviews, focus groups, observations, participatory approaches, etc.)	10	8	2
8. Knowing how to code qualitative data	10	4	6
9. Knowing how to analyse qualitative data	10	5	5
10. Interpreting your own qualitative data from records or other findings	10	8	2
11. Using findings from analysing records or other data for planning projects	10	6	4
12. Communicating your research findings effectively	10	6	4

The post-course survey showed a small, or no, gap between most research tasks participants considered very or moderately important, and their self-assessed ability to perform them well, or very well. The results indicated no gap in developing an appropriate research objective, designing research projects, identifying ethical concerns and communicating research findings.

**Chart 1.**
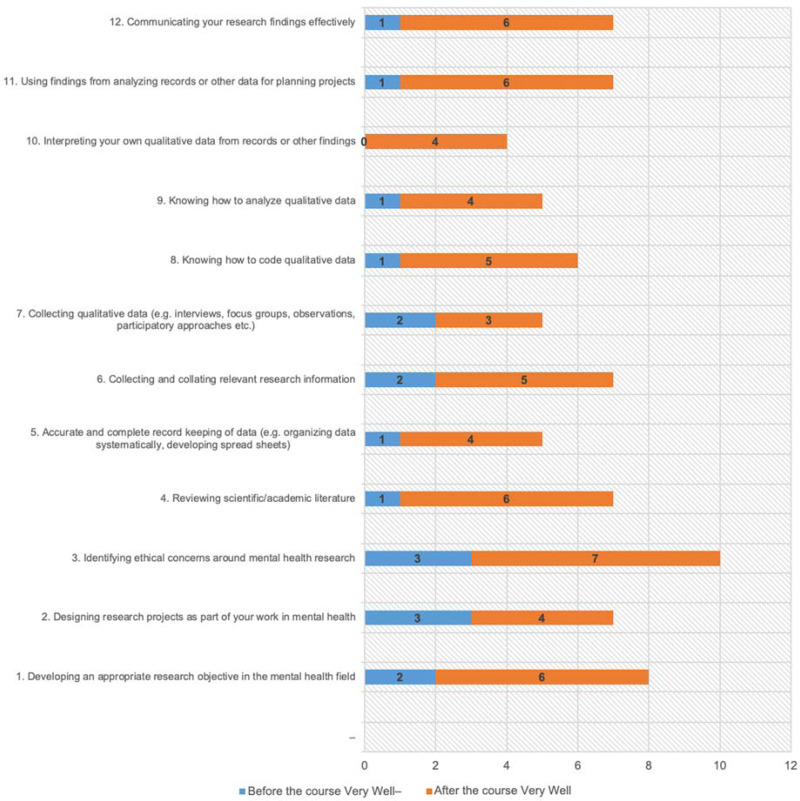
A Comparison Between Pre- and Post- Course Questionnaires. How well participants thought they could carry out a particular activity before and after the training.

Later, we compared the pre- and post-course surveys. The findings showed a tangible improvement in the participants' reported qualitative research skills. The number of participants who indicated their ability to perform all the research activities very well after the course was consistently higher than those who indicated a shortfall before the course. For example, before the course, only two participants noted they could develop an appropriate research objective very well, six well and two moderately well, while after the course, six noted very well and eight well (Chart 1).

### Course delivery evaluation

**Chart 2.**
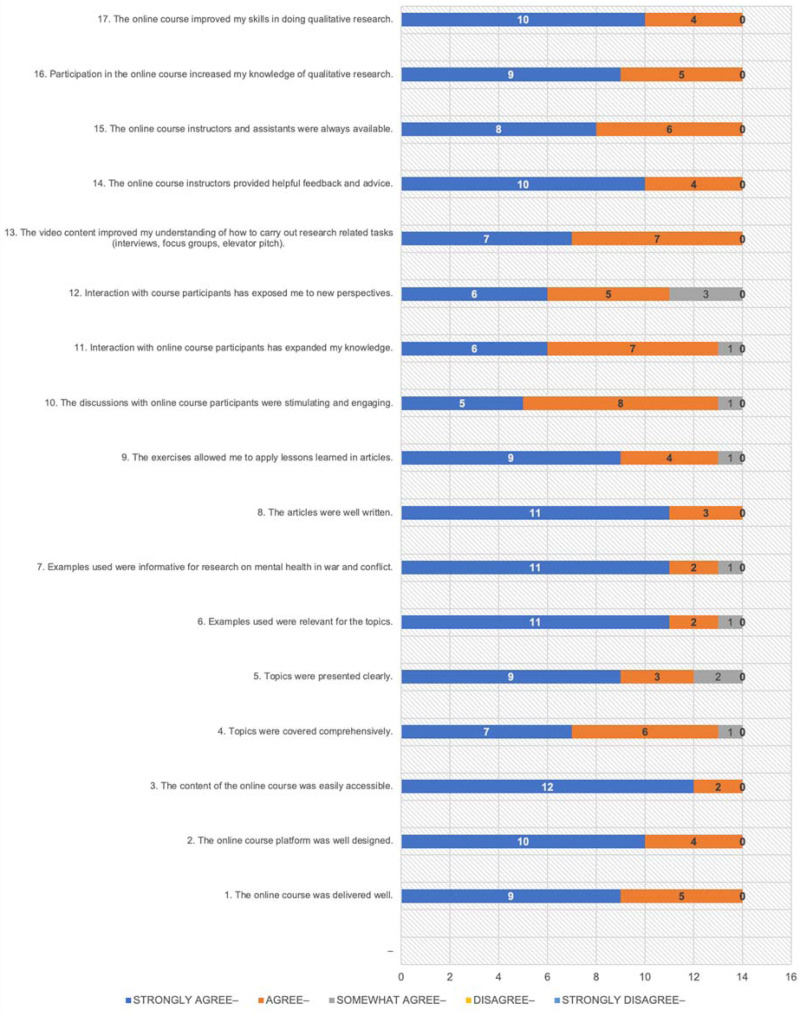
Course Delivery Evaluation.

All participants were highly satisfied with the course. Of the 14 who completed the evaluation, 10 strongly agreed, and four agreed that the course improved their qualitative research skills. The majority strongly agreed or agreed that the course was delivered well, that topics were covered comprehensively, that examples were relevant and informative, that discussions were stimulating, that exercises improved learning, and that the interaction with course participants expanded their knowledge (Chart 2).

### Qualitative course evaluation feedback and semi-structured interviews

Themes that were identified based on our thematic analysis included (1) the course was locally contextualised and met students' needs; (2) the course fostered dialogic and multi-directional learning and (3) suggestions for improvements. Notably, participants did not explicitly talk about, or criticise, the imposition of Western-centric epistemology, unidirectional knowledge transfer, neglect of local paradigms and notions of expertise. This may have been because the open-ended survey questions did not include probes into these topics and the semi-structured interviews were conducted with highly satisfied participants, who felt the course had catered to their expectations and needs.

#### Theme 1: the course was locally contextualised and met students' needs

Participants praised the accessibility of the course for Gazans who rarely get the opportunity to attend international training. One interviewee said, ‘There is no way to go out to travel, to communicate with people like you, so you are coming to us through these courses, so I do appreciate it’. Others praised the local relevance of the course. For example, one interviewee said:

This course was exactly what we needed for researchers in Gaza, we have so many conditions like wars, conflicts, mental health issues, but we need to know how to correctly do research, [and] how to collect data regarding these issues specifically. It's not like a general issue, it's like unique issues in Gaza, so I guess this course exactly touches on what we need.

Some participants commented that studies from other conflict regions had enriched their knowledge and widened their perspectives. One interviewee said, ‘With Palestinians, with refugees in Syria and other countries, we have some things in common, but when other examples like in Africa or in other countries, it added to my knowledge’.

Overall, participants found the course coherent, comprehensive and ‘full of valuable details’. Participants especially praised the diverse training methods. One interviewee said, ‘This course was one of the most amazing online training I had’, and described applying what she had learned during the course to improve her Master's research project: ‘I changed some of the techniques I was applying when I was collecting data based on the information provided in this course’. Other participants emphasised the course taught transferrable skills applicable to their jobs, which involve monitoring, evaluation and need assessment. One said: ‘It provides me with new knowledge and skills regarding the qualitative research theories, coding, analysis and findings presentation’.

Regarding technological aspects, accessibility and flexibility of the course delivery, participants liked our video content and, in particular, the FGD video produced at BZU that touched on the challenges people face in GS. It was also positively noted that the course format provided ‘the possibility of studying at any time’ and that the material was downloadable to access during electricity cuts. Only a few anticipated more advanced training, but confirmed the course consolidated their existing skills. For the majority, however, the course filled a gap in their qualitative capabilities.

#### Theme 2: the course fostered dialogic and multi-directional learning

Participants praised the interactive element with other participants and educators which they considered ‘very effective’. They appreciated the opportunities to participate and did not feel they were ‘at the receiving end’. During a semi-structured interview, one participant said, ‘Encouragement for discussions among participants inspired me to participate and interact’. Another interviewee praised the opportunity to initiate debates. Another participant said,

I can't say it is one direction, you used many of the technology or many of the skills, or the methodology, I guess two ways, because you were interactive, you are not just giving us the course online and not answering and submitting only. Through that course, we had discussions and what I appreciate that every comment we gave I found someone is answering my comment and discussing it with me.

Participants also liked the mix of local and non-local educators as it enriched their knowledge of other cultures and experiences.

From a critical perspective, a few participants were disappointed by the lack of engagement by some fellow participants. While acknowledging this could be due to work-life commitments, they proposed incorporating live components, or stringent enrolment criteria, with mandatory participation. They considered that exchanging experiences was a vital part of learning.

#### Theme 3: suggestions for improvements

While some participants found the course ‘easy’, others found several new topics challenging, such as how to define a relevant sample, coding and extracting themes. One participant described some of the content as ‘heavy meal of information [that] need[s] time to digest’. Other participants suggested the course run for longer, with more practical examples. One interviewee preferred to do more research tasks and critical analysis rather than quizzes, and suggested ending the course with a research project to present at a follow-up course. A few also wanted advanced training in topics such as participatory approaches and mixed methods. Several participants proposed conducting research supervised by course instructors, highlighting that research collaboration with Western institutions facilitates global visibility of local researchers. Participants suggested incorporating more interactive elements such as ‘online discussion’, ‘open meeting with trainers’ and ‘webinar sessions’. Finally, although the majority did not consider the English language a personal obstacle, with a few highlighting the vitality of English competency to reach the global audience, it was noted that ‘some highly qualified professionals might find the English language an obstacle’, and suggested providing course materials in the Arabic language.

## Discussion

Our online course aimed to strengthen the qualitative research capacities of MHPR in the GS to empower them to conduct high quality and locally relevant mental health research. To our knowledge, it is the first training delivered in the GS combining the fields of mental health, qualitative research methods and conflict, while addressing the ‘digital neo-colonialism critique’ (Binka, 2005; de Sousa Santos, 2015; Adam, 2019; Gallagher and Knox, 2019; King *et al*., 2019).

Our pedagogical practice was shaped by the awareness of inherent inequalities in global health research related to unequal access to resources, scientific information, high-quality training and opportunities for knowledge dissemination (Minn, 2015; Murphy *et al*., 2015). It was also informed by critical pedagogy which exposes the domination and propagation of Western knowledge generally (Freire, 1984; Thiong'o, 2005; Fanon, 2008), as well as in the digital realm of online training courses more specifically (Adam, 2019). In order to destabilise hegemonic practices to knowledge generation and dissemination, we tried to create spaces for a plurality of knowledges, experiences and histories to co-exist (Adam, 2019).

To achieve this, we employed a combination of methods for course development, content and delivery which enabled multi-directional learning, and integration of local paradigms and expertise (de Sousa Santos, 2015; Adam, 2019; Gallagher and Knox, 2019; King *et al*., 2019). Firstly, the course was the result of a longstanding collaboration between researchers from KCL and BZU, and the course materials were developed equally by a team of academics, research assistants, students and online technicians from both institutions. It has been shown that creating equal partnerships between academic institutions in HICs and LMICs is the optimum approach to ensure sustainable academic relationships, and to strengthen research capacities of new generations of local health professionals (Lansang and Dennis, 2007; Gezmu *et al*., 2011; Fricchione *et al*., 2012; Thornicroft *et al*., 2012; Semrau *et al*., 2018).

Secondly, our course content addressed a locally identified need for qualitative research training among MHPR (Giacaman *et al*., 2018). We incorporated relevant local and regional case studies, and accounted for the logistical challenges relating to electricity and Internet broadband.

Thirdly, our delivery fostered interaction, engagement, active and multi-way learning (Mason, 2003; International Telecommunication Union, 2006; Zander *et al*., 2006; Aczel *et al*., 2008; Dodani *et al*., 2012; Zaheer *et al*., 2015) by encouraging participants to share their expertise and local experiences, and by critically reflecting on the applicability of Western concepts and paradigms to their local realities. We also promoted dialogic and open discussions among participants and educators, with the option to communicate in Arabic and English. While such pluralistic and multi-directional learning has been shown to improve cultural competencies, research design and methods (Murphy *et al*., 2015), we were conscious that our training material itself was not as diverse. Even if mostly authored by scholars from the MENA region, it was heavily informed by Western approaches to knowledge generation and interpretation and, thus, not free from neo-colonial agendas which emphasise Western modes of knowing and suppress, or marginalise, indigenous ways of organising and conceptualising reality (Thiong'o, 2005; Minn, 2015; Adam, 2019). Our course is one step towards decolonial approaches that should involve more systemic and broader level change across the board.

Despite these limitations, the overall course feedback showed the course was well-received, leading to reported improvement in qualitative research skills. Participants felt their experiences, voices and local knowledge were incorporated and valued. Overall, the main strength of our course was its local relevance and contextualisation. However, course participants also highlighted course limitations and suggested improvements. They strongly highlighted that they would have liked to explore their different viewpoints and expertise in greater depth with each other and the course organisers via video conferencing platforms. We agree such an exchange could have led to more effective multi-way learning, critical reflections and ongoing suggestions for course improvement.

Other specific practical issues warrant reflection when developing similar training. Firstly, due to the large number of needs identified, only those of the highest demand, and essential to research, could be addressed in our course. Although few participants asked for specific topics to be taught and for others to be expanded, several found the content dense. To meet both requirements, further advanced courses could be designed building on this course and incorporating lessons learned. For future online courses, we recommend providing less dense content and offering follow-up opportunities combined with synchronously delivered drop-in sessions.

Secondly, we became aware of the huge costs and administrative challenges (Leary and Berge, 2006) because the material had to be developed specifically for the course, not simply converting traditional learning material to online material; so, we recommend designing material specific to the targeted audience, which combines a plurality of local and international epistemologies and case studies. Although our team reviewed course material, we recommend an independent reviewer from the respective conflict region to read the material critically.

Thirdly, since we believe evidence-based capacity strengthening is needed for mental health system strengthening (Hanlon *et al*., 2018), we attempted to capture the immediate outcome of our course by conducting pre- and post-course assessments and interviews with participants. However, due to budget, resource and timeframe restrictions, it is difficult to measure changes in capacity, and its impact, in the longer term (Vallejo and Wehn, 2016). Although our interviews showed a short-term positive impact, we stress the need for multi-path approaches to capture capacity changes and to evaluate their sustainability and impact over the medium, or long term (Vallejo and Wehn, 2016; Bowsher *et al*., 2019). Positive long-term impact would be course participants leading on publications, grants and academic production, based on locally identified needs, and carried out through locally developed research designs, methods and modes of interpretation (McKee *et al*., 2012; Bowsher *et al*., 2019; Sukarieh and Tannock, 2019).

Fourthly, to develop and implement courses like ours, it is important to ensure sustainability through funding, collaboration, research output and policy impact (Aczel *et al*., 2008; Siriwardhana *et al*., 2011; Vallejo and Wehn, 2016; Franzen *et al*., 2017). As our funding is time limited, we will have to make the course fully open access and sign some of our rights over to the FL platform for it to continue. The course will therefore become more commercial (while participants will be able to access learning material for free, they will have to pay for the course certificates), and, consequently, inequalities arise that are not in line with our pedagogical stance.

Overall, the development and implementation of this course was as much a learning experience for us as course developers, as it was for our participants. We became aware of the practical difficulties our learners face on the ground when conducting research, the limited and short-term learning opportunities, and the lack of continuous professional development to meet their needs. We were impressed by the resilience our participants showed, and their determination to produce high-quality local research, committed to exposing the living experiences and suffering of Gazans through contextually relevant frameworks that require critical engagement with theory and method. Although we do not claim to have overcome epistemic oppression, and are a long way from decolonising knowledge production and dissemination, we were pleased that the multi-directional nature of learning on our course allowed participants and instructors to swap roles at any point, and pushed against the assumed confines and directionality of some capacity strengthening models, whilst overcoming geopolitical borders imposed by military occupation and siege.

This course exemplifies early attempts to address the domination of Western knowledge in the developing world, including the oPt. Much needs to be done, given that educational and health systems are still largely dominated by Western epistemology, approaches and knowledge production. While this is the legacy of colonialism and post colonialism, it indicates the need to address the problem not only from a one-off training course, but also by systematically examining educational and health systems, working towards equalising power relationships, and relating epistemologies and knowledge to their relevance and appropriateness to the local context.

To this end, this project represents the beginning of what should be an ongoing project, seeking to make systems approaches more relevant and appropriate to context. Working in a settler colonial context, we are deeply aware that the hegemony of the Israeli state over the Palestinian society transcends to the academic arena, makes decolonising knowledge production a constant struggle for local and non-local researchers. Hence the importance of our course and similar efforts to strengthen research capacity with the vision of resisting the repression of indigenous knowledge and experience while building equitable international research partnerships. It is also essential to enhance the capacity and seek to employ local expertise at different levels, such as educators and technology-enhanced trainers and providers.

## Conclusions

In this article, we shared our experience of providing an online qualitative research method course, designed and delivered equally by KCL and BZU, for MHPR in GS. We described methods employed to address digital neo-colonialism, and presented findings of the pre-course need assessment, post-evaluation and semi-structured interviews. To achieve sustainable and locally responsive online research capacity in conflict settings, a plurality of voices, histories and epistemologies must be valued and integrated. Our future outlook is to improve the online course. We will reduce the density of the content, include live, interactive sessions, and provide the course content in Arabic and English. While doing so, we aim to foster multi-directional learning to ensure that non-Western knowledge, epistemologies and languages gain a prominent presence in the Western academic world. We believe a real social transformation would see local researchers and educators confidently engage with, and use, local methods and paradigms in the international realm as they design projects, collect data and develop theories for mental health in war and conflict.

## Appendix 1

The pre-course questionnaire

**Table:**

RCUK online course participant baseline questionnaire	
Personal information	
Q1	Gender:
	Male
	Female
Q2	What is the highest educational degree you have received?
	Undergraduate (B.Sc. or B.A.)
	Master (M.A. or M.Sc.)
	Ph.D.
	Other, specify: _________________________________
Institutional information	
Q3	8.1. Type of institution:
	Governmental
	UNRWA
	Local NGO
	International NGO
	Other, specify: ______________________________
	8.2. How would you describe your institution's main focus? *Circle as many as applicable*
	Education
	Research
	Service
	Community development
	Other; specify:
Q4	How many years have you been working at this institution?
	|__|__|
Research activities and training	
Q5	21.1. Are you involved in some form of data management (i.e. beneficiary record keeping, analysis of beneficiary data)?
	Yes
	Yes sometimes
	No
Q6	Are you involved in any of the following activities? *Circle as many as applicable*
	Projects development
	Surveys
	Interviews
	Observations
	Report writing
	Research projects
	Other, specify: ___________________________
Course expectation	
Q7	What do you expect to learn from this course?
	How do you hope to be able to apply the newly-gained knowledge in your work?

Training questionnaire Please read the two questions (A and B) and circle the answer:										
	Question A					Question B				
	How important is this activity for you and/or your job?					How well do you think you can carry these activities out?				
	Not at all	A little	Moderately	Important	Very important	Not at all	A little	Moderately	Well	Very well
Research project development										
1. Developing an appropriate research objective in the mental health field	1	2	3	4	5	1	2	3	4	5
2. Designing research projects as part of your work in mental health	1	2	3	4	5	1	2	3	4	5
3. Identifying ethical concerns around mental health research	1	2	3	4	5	1	2	3	4	5
Research methods										
1. Reviewing scientific/academic literature	1	2	3	4	5	1	2	3	4	5
2. Accurate and complete record keeping of data (e.g. organising data systematically, developing spread sheets)	1	2	3	4	5	1	2	3	4	5
3. Collecting and collating relevant research information	1	2	3	4	5	1	2	3	4	5
4. Collecting qualitative data (e.g. interviews, focus groups, observations, participatory approaches, etc.)	1	2	3	4	5	1	2	3	4	5
Data analysis										
1. Knowing how to code qualitative data	1	2	3	4	5	1	2	3	4	5
2. Knowing how to analyse qualitative data	1	2	3	4	5	1	2	3	4	5
Application and dissemination										
1. Interpreting your own qualitative data from records or other findings	1	2	3	4	5	1	2	3	4	5
2. Using findings from analysing records or other data for planning projects	1	2	3	4	5	1	2	3	4	5
3. Communicating your research findings effectively	1	2	3	4	5	1	2	3	4	5

## Appendix 2

The post-course questionnaire

**Table:**

**RCUK** qualitative online course evaluation	
Personal information	
Q1	Gender: 1. Male 2. Female
Q2	What is the highest educational degree you have received? 1. Undergraduate (B.Sc. or B.A.) 2. Master (M.A. or M.Sc.) 3. Ph.D. 4. Other, specify: _________________________________
Institutional Information	
Q3	8.1. Type of institution: 1. Governmental 2. UNRWA 3. Local NGO 4. International NGO 5. Other, specify: ______________________________ 8.2. How would you describe your institution's main focus? *Circle as many as applicably* 1. Education 2. Research 3. Service 4. Community development 5. Other; specify:
Q4	How many years have you been working at this institution? |__|__|
Research activities and training	
Q5	21.1. Are you involved in some form of data management (i.e. beneficiary record keeping, analysis of beneficiary data)? 1. Yes 2. Yes sometimes 3. No
Q6	Are you involved in any of the following activities? *Circle as many as applicable* 1. Projects development 2. Surveys 3. Interviews 4. Observations 5. Report writing 6. Research projects 7. Other, specify: ___________________________

Training questionnaire Please read the two questions (A and B) and circle the answer:
	Question A	Question B
	How important is this activity for you and/or your job?	How well do you think you can carry these activities out?
	Not at all	A little	Moderately	Important	Very important	Not at all	A little	Moderately	Well	Very well
Research project development										
1. Developing an appropriate research objective in the mental health field	1	2	3	4	5	1	2	3	4	5
2. Designing research projects as part of your work in mental health	1	2	3	4	5	1	2	3	4	5
3. Identifying ethical concerns around mental health research	1	2	3	4	5	1	2	3	4	5
Research methods										
4. Reviewing scientific/academic literature	1	2	3	4	5	1	2	3	4	5
5. Collecting and collating relevant research information	1	2	3	4	5	1	2	3	4	5
6. Collecting qualitative data (e.g. interviews, focus groups, observations, participatory approaches, etc.)	1	2	3	4	5	1	2	3	4	5
Data analysis										
7. Knowing how to code qualitative data	1	2	3	4	5	1	2	3	4	5
8. Knowing how to analyse qualitative data	1	2	3	4	5	1	2	3	4	5
Application and dissemination										
9. Interpreting your own qualitative data from records or other findings	1	2	3	4	5	1	2	3	4	5
10. Using findings from analysing records or other data for planning projects	1	2	3	4	5	1	2	3	4	5
11. Communicating your research findings effectively	1	2	3	4	5	1	2	3	4	5

Course delivery evaluation					
	Strongly agree	Agree	Somewhat agree	Disagree	Strongly disagree
1. The online course was delivered well.	1	2	3	4	5
2. The online course platform was well designed.	1	2	3	4	5
3. The content of the online course was easily accessible.	1	2	3	4	5
4. Topics were covered comprehensively.	1	2	3	4	5
5. Topics were presented clearly.	1	2	3	4	5
6. Examples used were relevant for the topics.	1	2	3	4	5
7. Examples used were informative for research on mental health in war and conflict.	1	2	3	4	5
8. The articles were well written.	1	2	3	4	5
9. The exercises allowed me to apply lessons learned in articles.	1	2	3	4	5
10. The discussions with online course participants were stimulating and engaging.	1	2	3	4	5
11. Interaction with online course participants has expanded my knowledge.	1	2	3	4	5
12. Interaction with course participants has exposed me to new perspectives.	1	2	3	4	5
13. The video content improved my understanding of how to carry out research related tasks (interviews, focus groups, elevator pitch).	1	2	3	4	5
14. The online course instructors provided helpful feedback and advice.	1	2	3	4	5
15. The online course instructors and assistants were always available.	1	2	3	4	5
16. Participation in the online course increased my knowledge of qualitative research.	1	2	3	4	5
17. The online course improved my skills in doing qualitative research.	1	2	3	4	5

What did you particularly like about the course?

What did you dislike about the course?

Were there any components of the course that you found challenging to understand?

Were there any topics that you wished to be covered and were not included in the course? If yes, please list them

How do you think the course could be improved?

What kind of follow-up training would you like to receive?

## Appendix 3

Interview guide:

Name:

Gender:

Date of birth

Professional affiliations:

Job title:

1. Please read the following statement to discuss in link with our course. “Research capacity in conflict environments is characterized by unidirectional knowledge transfer, imposition of Western knowledge, and neglect of local expertise”.
2. Do you think your learning experience would have been different if the course had been designed and delivered either (a) only by Palestinian researchers and teachers or (b) only British researchers and teachers?
3. How sensitive is the training course to the needs and skills of the participants? Please think about the positive and negative aspects.
4. To what extent does the course value and integrate the participants' knowledge and experience? Please think about the positive and negative aspects.
5. What are your thoughts on the case studies that were used in the course? Do you feel that they reflect the local and regional context in which you live and work? If so, how? If not, why not?
6. How successful was the course in stimulating ideas about local concepts and experiences? Please think about the positive and negative aspects.
7. What do you think about the fact that the course content and exercises are written in English? Do you think that your participation would have been different if the course was in Arabic? If so, why?
8. Were you able to use any part of the course material, conceptual, methodological or other, in your work/research/studies? Usage does not necessarily mean only systematic or practical, but can also mean how to visualize problems in different ways compared to before taking the course?
9. In your opinion, how can the course be improved to be more responsive to local needs and aspirations?
10. Is there anything else you would like to add?

1) يرجى قراءة  المقطع  التالي والتفكير فيه فيما يتعلق بدورتنا: في  الأبحاث  قيل أن "التعلم عبر الإنترنت المستخدم لتعزيز ". القدرة البحثية في بيئات الصراع يتميز بنقل المعرفة أحادي الاتجاه ، وفرض المعرفة الغربية ، وإهمال الخبرة المحلية مناقشة

2) هل تعتقد أن تجربة التعلم الخاصة بك كانت ستختلف لو تم تصميم الدورة وتقديمها إما (أ) فقط من قبل الباحثين والمدرسين الفلسطينيين أو (ب ) من قبل الباحثين والمعلمين البريطانيين فقط؟

3) ما مدى مراعاة الدورة التدريبية لاحتياجات المشاركين ومهاراتهم؟ يرجى التفكير في الجوانب الإيجابية والسلبية.

4) إلى أي مدى تقدر الدورة وتدمج معارف وخبرات المشاركين؟ يرجى التفكير في الجوانب الإيجابية والسلبية.

التي تم استخدامها في الدورة؟ هل تشعر أنها تعكس “case studies” 5) ما هي أفكارك حول دراسات الحالة السياق المحلي والإقليمي الذي تعيش فيه وتعمل فيه؟ إذا كان الأمر كذلك ، فكيف؟ إذا لم يكن كذلك ، فلماذا لا؟

6) ما مدى نجاح الدورة في تحفيز الأفكار حول المفاهيم والتجارب المحلية؟ يرجى التفكير في الجوانب الإيجابية .والسلبية

7) ما رأيك في حقيقة أن محتوى الدورة والتمارين مكتوبة باللغة الإنجليزية؟ هل تعتقد أنك مشاركتك كانت ستختلف لو كانت الدورة باللغة العربية؟ إذا كان الأمر كذلك، لماذا؟

8) هل تمكنت من استخدام أي جزء من مادة الدورة التدريبية ، المفاهيمية أو المنهجية أو غيرها ، في عملك / أبحاثك / دراساتك؟ لا يعني الاستخدام بالضرورة منهجيًا أو عمليًا فحسب ، بل يمكن أن يعني أيضًا كيفية تصور المشكلات بطرق مختلفة مقارنةً بما قبل أخذ الدورة؟

9) في رأيك ، كيف يمكن تحسين الدورة لتكون أكثر استجابة للاحتياجات و التطلعات المحلية ؟

10) هل هناك أي شيء آخر تود إضافته؟
