# Towards decolonising research methods training: The development of a locally responsive online learning course on research methods for mental health in war and conflict for researchers and practitioners in the Gaza Strip – ERRATUM

**DOI:** 10.1017/gmh.2021.46

**Published:** 2021-12-09

**Authors:** Nancy Tamimi, Hanna Kienzler, Weeam Hammoudeh, Hala Khalawi, Mathias Regent, Rita Giacaman

The publisher apologises that upon publication of this article the authors names were swapped around, presenting the surname as their first.

The authors correct names are Nancy Tamimi, Hanna Kienzler, Weeam Hammoudeh, Hala Khalawi, Mathias Regent and Rita Giacaman

The online version of this article has been updated.
